# The Roles of Endorsement and Stigma in Suicidal Ideation and Behavior among Chinese College Students

**DOI:** 10.3390/ijerph20010877

**Published:** 2023-01-03

**Authors:** Shunyan Lyu, Yu Li

**Affiliations:** Department of Life Sciences, BNU-HKBU United International College, Zhuhai 519087, China

**Keywords:** suicidal ideation, suicide attempts, stereotypes, stigma, college students

## Abstract

Previous studies have suggested that stereotypes towards suicide, including endorsement of suicide and stigma toward suicide, may contribute to suicidal ideation and behaviors. However, this has not been examined directly. In this study, we examined whether endorsement of suicide and stigma toward suicide are involved in the pathway from suicidal ideation to suicide attempts among college students. To this end, we used the Suicidal Ideation Attributes Scale (SIDAS), the Suicidal Behaviors Questionnaire-Revised (SBQ-R), and the Stigma of Suicide Scale (SOSS) to assess suicidal ideation, suicide attempts, endorsement of suicide, and stigma toward suicide, respectively, in a sample of 944 Chinese college students (mean age, 20.97 years). Using mediation analysis, we found that suicidal ideation partially mediated the relationship between endorsement of suicide and suicide attempts and between stigma toward suicide and suicide attempts. These findings provide novel evidence that endorsement of suicide and stigma toward suicide are closely associated with suicide attempts, but partially through the influences of suicidal ideation. Future studies should elaborate on their longitudinal relationships. Implications of these findings for clinical practices are discussed with reference to the ideation-to-action framework of suicide, aiming to reduce suicidal behaviors.

## 1. Introduction

Suicide is the act of deliberately killing oneself [1]. Suicide has received significant attention from the World Health Organization (WHO) and additional medical fields [2]. A recent report issued by WHO showed that suicide rates globally were roughly 9.2 per 100,000 in 2019, and the suicide rate among men was more than twice that among women in that year (12.6 per 100,000 for men and 5.7 per 100,000 for women) [3]. Suicide rates in China are consistently lower than at international levels. However, the China Health Statistic Yearbook 2021 presented some worrying data on suicide rates [4]. In 2020, the suicide rate was 4.58 per 100,000 in urban areas and 7.47 per 100,000 in rural areas [4], and all these rates were higher than the 2019 rates [5]. For both urban and rural areas, the 2020 suicide rate was higher in men than in women [4], and again all rates were higher than they were in 2019 [5]. Specifically, for young Chinese people aged 15–25 years, the 2020 suicide rate was higher in men than in women [4]. For urban populations of both genders, the 2020 suicide rate was the highest from 2011 to 2021 [4,5,6]. Furthermore, the suicide rates of younger cohorts (15–35 years old) tended to become higher than that of the groups aged less than 45 years [4]. According to existing studies, suicide is one of the major causes of death for 15- to 35-year-olds in Chinese society [7]. In this population, college students have a higher suicide rate than other age groups due to psychiatric and social issues (e.g., mental health, academic pressure, economic pressure, family education, negative life events, media, and social platforms) [8,9]. Suicide in these groups should receive more attention.

According to Beck [10], there are three key concepts on which the study of suicide should be based: suicidal ideation, suicide attempts, and completed suicide. When thoughts or ideas of suicide are generated but no motivational process occurs, the individuals are said to have suicidal ideation. When there is a motivational but incomplete process, the individuals are said to have suicide attempts [11]. Suicidal ideation is usually a necessary premise of suicide attempts, although no direct evidence could support the causal relationship between them [12,13].

According to the ideation-to-action framework in the three-step theory proposed by Klonsky and May [13,14], the first step toward suicide is the development of suicidal ideation, for which pain and hopelessness are needed. The second step is the formation of strong suicidal ideation, which is a status with the decreasing or collapsing of “connectedness” including social connection, sense of meaning or purpose, and the feelings of being a part of society. The third step is suicide attempts, during which individuals have the capability for suicide (including dispositional, acquired, and practical capability).

A variety of influencing factors (e.g., psychological pain, hopelessness, and mental diseases) can be considered when determining suicidal ideation in the ideation-to-action framework [15]. One of these factors is stereotypes toward suicide (e.g., endorsement of suicide and stigma toward suicide), which has received less attention than other easily measurable factors such as academic pressure and economic problems [9,16,17,18,19]. Stereotypes toward suicide are a series of incomplete, labeled perceptions of persons having suicide-related behaviors. Research on stereotyping commonly focuses on topics of prejudice, discrimination, intergroup relations, and stigma [20,21]. In the current study, we focused on positive stereotypes and negative stereotypes. According to existing studies, people who are more likely to identify with positive stereotypes toward suicide tend to recognize and attempt suicide [22]. Meanwhile, people who are more likely to accept negative stereotypes are more likely to resist suicidal behavior [23,24,25,26,27].

Endorsement of suicide represents a positive stereotype toward suicide. Two influential factors should be explicitly mentioned for a better understanding of college students’ endorsement of suicide in the context of Chinese culture. Endorsement of suicide is recommended in certain situations in Confucian societies [28]. For example, people who conduct suicide for their country or family are likely to have fame and will be remembered by the following generations [29]. This cultural tradition is believed to strengthen Chinese people’s endorsement of suicide, which has also been found to increase suicide rate, as indicated in some studies [30]. In addition to culture, social media is a crucial source of endorsement of suicide. Media and social platforms are widely and conveniently accessed by young generations, both in Chinese societies and other countries [31,32,33]. Some studies have examined the roles of public media in influencing audiences into mimicking suicide after highly publicized incidences of suicide, which is known as the Werther effect [34]. People are tragically led by highly publicized suicidal stories and then try to conduct suicide by imitation [35,36]. The increasing popularity of social platforms raised concerns about suicide contagion, and the mechanism of suicide contagion is thought to be based on social learning [37]. The platforms are an important medium for people who have suicidal ideation to conveniently communicate with each other [38]. People who have suicidal ideation may share detailed information on suicide and imitate similar behaviors, which exacerbates suicidal behaviors [37,39,40,41].

Stigma toward suicide represents a negative stereotype of suicide. Erving Goffman first used stigma to describe social members who were socially “abnormal”. Researchers have proposed different classifications for stigma [42,43]. Stigma can be classified into two types: public stigma and self-stigma [44]. Public stigma refers to the words or actions of the majority with prejudice toward minority groups in society, and self-stigma is identifiable in the members of a stigmatized group who proactively accept these prejudgments [45]. The former usually occurs first with false allegations and labels from others that are then internalized by victims, followed by self-stigma which may threaten personal mental health [43,46]. This phenomenon is found to be much more common in people with mental illness, who have a significantly high rate of suicidal ideation [31,47]. However, Corrigan and colleagues [48] suggested that self-stigma could occur without public stigma, although both are caused by blame, dangerousness, and incompetence. Some researchers suggest that stigmatizing attitudes toward suicide could also be rooted in culture. Many college students have stigmas toward both mental illness and suicide, and they tend to feel shameful about seeking psychological counseling and be less reachable by intervention [9,43]. However, there is also evidence that negative attitudes toward suicide could increase individuals’ willingness to find help and suppress suicide attempts [22,23]. The differences between self-stigma and public stigma may explain these two seemingly opposing phenomena. Self-stigma toward suicide likely shares similar mechanisms to self-stigma toward mental illness. People who have internalized such kinds of self-stigma tend to agree with others who hold unwarranted and unrealistic prejudices. On the contrary, people who have highly recognized public stigma toward suicide likely try to suppress their suicide attempts, although this is unlikely to eliminate suicidal ideation completely.

Based on the literature reviewed above, existing studies considered social and cultural factors of suicide as crucial as other risk factors [8,9,49]. Endorsement of suicide is greatly influenced by these two interacting factors, and stigma toward suicide was closely related to social factors [28,29,30,31,32,33]. Therefore, we predict that endorsement of suicide and stigma toward suicide are indirectly associated with suicidal ideation and behavior. If these relationships can be demonstrated in a population of Chinese college students, it will be a significant contribution to both suicide-related education and suicide prevention in college students (and possibly the general population). However, the relationships between endorsement of suicide, stigma toward suicide, suicidal ideation, and suicide attempts remain unknown. More specifically, it is unclear whether and how endorsement and stigma can be linked to the ideation-to-action framework [13,14,50,51]. The present study examines these questions in college students who show a high suicide risk. We hypothesized that endorsement of suicide and stigma toward suicide would have an influence on suicide attempts through suicidal ideation, which is considered the premise of suicide attempts. Furthermore, it has been reported that there are large gender differences in mortality from suicide [52,53], suicidal ideation [9], suicide attempts [6] (but see [54]), and stigma toward suicide [55,56]; however, gender effects on stigma toward suicide and the relationships among it, suicidal ideation, and attempts are still poorly understood. We therefore also explored these research questions.

## 2. Method and Materials

### 2.1. Participants

Participants were asked to complete questionnaires hosted on the online data collection platform Wenjuanxing (https://www.wjx.cn/ accessed on 29 December 2022). Data from 944 Chinese college students (60.38% women) were obtained for the final analysis. Table 1 provides participant distributions based on gender and educational level. All participants were Chinese citizens who were studying in colleges in China and aged 20.97 years (SD = 2.22). Most were from several colleges in Guangdong province located in southern China. Of the participants, 374 were men aged 20.97 years (SD = 1.66) and 570 were women aged 20.97 (SD = 2.52). The current study was approved by the Research Ethics Committee of BNU-HKBU United International College.

### 2.2. Procedure

All the questionnaire data were collected using snowball sampling. Participants completed electronic questionnaires by scanning a QR code generated by Wenjuanxing. Prior to the data collection, electronic informed consent forms were obtained from participants. For those who wanted to keep a paper-based form for themselves, we also provided them paper-based informed consent forms with a handwritten signature. In the data collection, participants provided demographic information pertaining to gender, age, and education level, and then completed three scales, namely the Stigma of Suicide Scale—Short Form, Suicidal Ideation Attributes Scale, and Suicidal Behaviors Questionnaire-Revised. No personal information, including name, student ID, and residential address, was required from participants. After the data collection, participants were given the contact phone numbers of national and school mental health services for those who were seeking help and support. Moreover, personal information could not be traced via the QR code.

### 2.3. Measures

The Stigma of Suicide Scale (SOSS) is a five-point Likert scale for assessing stigmatizing attitudes [57]. It consists of three subscales: the stigma subscale, the isolation/depression subscale, and the glorification/normalization subscale [58]. The SOSS is scored by calculating three separate scores by adding up all the items in the subscales, respectively. In the current study, we used glorification/normalization and stigma. A higher score of glorification/normalization indicates a stronger endorsement of suicide, and a higher score of stigma indicates a stronger stigma toward suicide. Acceptable reliability and validity coefficients of this scale have been reported among college students from four countries, including China (Cronbach’s α is above 0.72) [59]. In this study, we used a revised version of the Chinese SOSS (12 items), and Cronbach’s α values for the three subscales were 0.84 (stigma subscale), 0.83 (isolation/depression subscale), and 0.81 (glorification/normalization subscale), respectively.

The Suicidal Ideation Attributes Scale (SIDAS) was used to assess personal suicidal ideation [60]. This five-item scale measures properties of suicidal thoughts including frequency, controllability, closeness to attempts, level of distress associated with the thoughts, and impact on daily functioning. Participants rate themselves by selecting a number from 0 to 10 to evaluate the frequency of certain thoughts. All items are summed to generate the total score. Participants who selected “Never (0)” to the first item (“In the past month, how often have you had thoughts about suicide?”) automatically skipped the remaining four items and received 0 for the total score. A higher score means a higher level of suicidal ideation. A total score greater than 20 indicates a high risk of suicidal behavior. This scale has displayed high internal consistency (Cronbach’s α = 0.91) and good convergent validity with other measures of suicide [58]. Cronbach’s α was 0.85 in the current study.

The Suicidal Behaviors Questionnaire-Revised (SBQ-R) was used to assess suicidal behavior [61]. This four-item questionnaire focuses on suicidal behavior in the past of individuals. Participants were instructed to choose one description that would best fit their situation in order to evaluate the frequency of certain behavior. Adding up all the items yields the total scores, and a higher score indicates a higher level of suicide attempts. A total score greater than 6 indicates a high suicide risk (i.e., “suicide attempters” in this study). The Chinese version of SBQ-R showed acceptable internal consistency (ρ = 0.79, ω = 0.75) [62]. Cronbach’s α was 0.77 in the current study.

### 2.4. Data Analyses

All data analyses were conducted using JASP (version 0.16.2), IBM SPSS 27.0 (IBM SPSS Inc., Chicago, IL, USA) and Process Macro (Hayes, 2012, white paper). Pearson correlation analyses were conducted to examine the correlations among stigma toward suicide, attribution of suicide to isolation/depression, glorification/normalization of suicide, suicidal ideation, and suicide attempts. Mediation analysis was used to determine if suicidal ideation served as a mediating variable in the relationship between glorification/normalization of suicide and suicide attempts. Participants’ gender, age, and education level were controlled for in the mediation analysis. A bootstrapping procedure (5000 times) was used to determine 95% confidence intervals of the mediating and direct effects. Furthermore, we examined gender differences in the correlations and mediating effects by using the same procedures. In these mediation analyses, age and educational level were controlled for as covariates.

## 3. Results

### 3.1. Suicide Risk

Table 2 provides the descriptive statistics of the variables. One-sixth of the participants (16.42%; *n* = 155) were at high risk of suicidal behavior (total SIDAS score ≥ 21). Of these participants, 25.16% (*n* = 39) had attempted suicide (SBQ-R Item 1 ≥ 3; “Have you ever thought about or attempted to kill yourself?”). In the total sample, 13.14% (*n* = 124) were at risk of suicide (total SBQ-R score ≥ 7), and 86.86% (*n* = 820) had not attempted suicide.

### 3.2. Correlations among Variables

Table 3 provides the intercorrelations among the variables. The results showed that glorification/normalization of suicide was significantly correlated with suicidal ideation and behavior, and suicidal ideation and behavior were significantly correlated (*r* = 0.49, *p* < 0.001). However, isolation/depression was significantly correlated with suicide behavior but not suicidal ideation.

### 3.3. Mediating Effect of Suicidal Ideation

**Figure 1:**
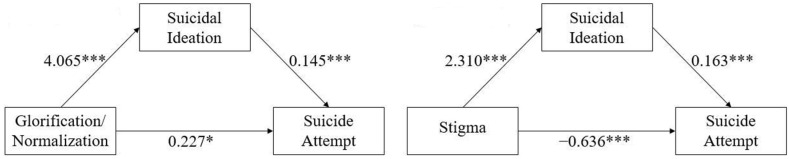
The mediation model of suicidal ideation in the association between glorification/normalization of suicide (**left**) and stigma toward suicide (**right**) and suicide attempts. * *p* < 0.05, *** *p* < 0.001.

Mediation analysis, with gender, age, and educational level being controlled for, revealed a significant mediating effect of suicidal ideation between glorification/normalization of suicide and suicide attempts (*p* < 0.001), as well as a significant direct effect (*p* < 0.022), indicating a partial mediating role of suicidal ideation. The mediating effect accounted for 72.20% of the variance in the total effect. Furthermore, we also found a significant mediating effect of suicidal ideation as the mediator between stigma toward suicide and suicide attempts (*p* < 0.001) as well as a significant direct effect (*p* < 0.001), indicating a partial mediating role of suicidal ideation. The mediating effect accounted for 59.08% of the variance in the total effect. The estimated direct, mediating, and total effects are shown in Table 4 and Figure 1.

### 3.4. Gender Comparison

We further conducted t-tests to determine the statistical significance of the gender differences in the scores of each measure (Table 5). We found that the SIDAS scores were higher in men than in women (*p* < 0.001), and the SOSS isolation/depression and SOSS stigma scores and SBQ-R scores were higher in women than in men (*p* < 0.001). No significant gender differences were found in the SOSS glorification/normalization scores (*p* = 0.084).

**Figure 2:**
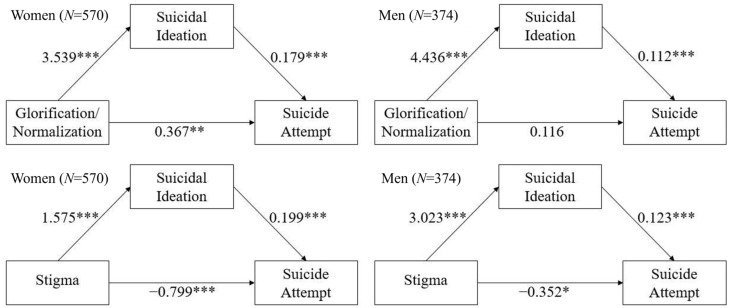
The results of the mediation analyses for glorification/normalization (**above**) and stigma (**below**) in women (**left**) and men (**right**). * *p* < 0.05, ** *p* < 0.01, *** *p* < 0.001.

We then conducted mediation analyses separately based on gender. These analyses again had age and educational level controlled for and were conducted for both glorification/normalization and stigma. The results first revealed a significant mediating effect of suicidal ideation as the mediator between glorification/normalization of suicide and suicide attempts in both groups (*p* < 0.001; Table 6, Figure 2). However, the direct effect was only significant for women (*p* = 0.006), not for men (*p* = 0.433). These results suggested a partial mediating effect in women (63.27% of the variance in the total effect) and a full mediating effect in men (81.21% of the variance in the total effect). Second, we found a significant suppression effect of suicidal ideation as the mediator between stigma toward suicide and suicide attempts in women (*p* = 0.002; Table 6, Figure 2). This suggested a partial mediating effect in women (39.23% of the variance in the total effect).

## 4. Discussion

Suicide is one of the biggest contemporary issues. Elucidating suicide-related issues helps us better understand the influencing factors of suicide and can help us propose effective preventive measures against suicidal behaviors. In the current study, we specifically examined the influences that endorsement of suicide (glorification/normalization of suicide) and stigma toward suicide have on suicide attempts as well as the mediating role of suicidal ideation among Chinese college students. Using simple mediation analysis, we found that the effect of endorsement on suicide attempts was partially mediated by suicidal ideation. This mediation was observed in both men and women, but men had a full mediating effect. We also found that the effect of stigma toward suicide on suicide attempts was partially mediated by suicidal ideation, but only in women. Furthermore, we observed that suicidal ideation was higher in men than in women, but suicide attempts were higher in women than in men. These novel findings are discussed below.

To our knowledge, the current study is among the first that is aimed at examining the relationships between endorsement and the ideation-to-action framework of suicide [14]. In western countries, researchers have already conducted a series of studies on stereotypes of suicide and the psychological factors in both clinical and community samples [57,63,64]. Notably, Han and colleagues conducted a cross-cultural study with Australian and Chinese college students, in which they specifically compared help-seeking and stigma toward suicide in the two populations [65]. However, none of these studies included suicide attempts in the suicidology research of stereotypes. Han and colleagues assessed suicidal ideation, stigmatizing attitudes toward suicide, and knowledge of suicide prevention in a sample of Chinese college students [59]. The results showed strong correlations among these variables and suggested that there was a lack of education on the topic of suicide in Chinese colleges. In a study assessing stigma toward suicide, suicide attempts, and suicide survival in a student sample, Wu and colleagues revealed positive correlations between suicidality, suicide exposure, and endorsement of suicide [66]. However, these studies mainly focused on scale development and translation and did not explore suicide attempts. According to Sun et al.’s study, both influencing factors and mechanisms of suicide are still poorly understood in the ideation-to-action framework of suicide [19]. Extending these contributions, the current exploratory study sought to understand suicidal ideation and suicide attempts, and specifically examined the possible relationship between the endorsement of suicide and the ideation-to-action framework.

In theory, stigmatizing attitudes that contribute to suicide risk consist of social and individual factors [11]. Negative attitudes could be reflected in public stigma, and they could also be internalized as negative attitudes that people have about their own condition, known as self-stigma [44,45]. Public stigma and self-stigma have some commonalities. These stigmatizing attitudes are based on misunderstandings and stereotypes, which usually lead to discrimination against others or oneself [48]. Fundamental differences also exist between the two types of stigmas. Self-stigma is usually caused by strong public stigma and victims’ internalization of stereotypes [43]. Vogel and colleagues suggested that the internalizing process of stigma, i.e., self-stigma, may make individuals with mental illness less willing to seek help or support [67]. The two types of stigmas reduce counseling-seeking behaviors and therefore increase the difficulty of suicide prevention efforts [46,58,68,69]. Naturally, it is more difficult for professionals to provide help to people who have strong self-stigma.

In the sample of the current study, especially among women, public stigma toward suicide inhibits suicide attempts, even though it does not inhibit suicidal ideation. These results show that stigma toward suicide has a more complicated mechanism through its link to the ideation-to-action framework of suicide. There are two possible paths for understanding these mechanisms. First, public stigma toward suicide possibly leads to internalized stigma (i.e., self-stigma) and then enhances suicidal ideation [43,46,69]. Second, perceived public stigma toward suicide increases the likelihood that individuals will seek help and suppress suicide attempts [22,23]. The two possible paths show converse effects, but likely coexist. The suppression effect observed in this study could be explained by the second path. The current study did not measure self-stigma, leaving an unaddressed question, namely whether public stigma leads to self-stigma, which in turn contributes to suicidal ideation and suicide attempts. To capture the wider picture of the influences of stigma on suicide, it is worthwhile to analyze both public stigma and self-stigma toward suicide. Further investigations addressing this question could significantly contribute to our understanding of how public stigma toward suicide is indirectly predictive of suicide.

Stereotypes toward suicide may be rooted in culture and social media [28,29,30,34,35,36,37]. The evidence linking stereotypes toward suicide and the ideation-to-action framework suggests potential suicide prevention strategies for suicide-related researchers and educators. The first recommendation is to provide education about suicide to college students [49]. Colleges can disseminate knowledge to form a better and more accurate understanding of suicide among college students, reducing false impressions of suicidal ideation and attempts. The existing mental health and education system in Chinese colleges could build campus-based programs that are available for college students [37]. The second recommendation is to prevent suicide through public and social media platforms [9,70]. The popularity of public and social media platforms leads to an increase in the stereotypes toward suicide. Governments and other non-government organizations should actively get involved in establishing platforms or holding various activities to reduce the spread of the information that endorses suicide.

According to the scoring criteria of SIDAS, 16.42% of the participants were identified as displaying suicidal ideation and were at high risk of suicidal behavior. It is difficult to compare these findings with early studies focusing on Chinese college students [8,71] because different scales for measuring suicide ideation were used. For example, Wu et al. [71] used Beck Scale for Suicide Ideation and found that 18% of college students showed high suicidal ideation, which is similar to the current study. Li et al. [8] meta-analyzed 41 studies involving 160,339 Chinese college students and found that the prevalence of suicidal ideation among the students was 10.72%. We can see an increase in ideation if we compare their percentage with ours. The increase may reflect a general trend over the past years in China and may reflect the long-term social and economic disruptions caused by the COVID-19 pandemic [72,73].

Regarding gender differences, our results showed that the SOSS isolation/depression and stigma were higher in women than in men. To our knowledge, this finding is the first of its kind to be reported. However, the reasons behind this gender difference are not clear. Future studies are needed to shed light on this. Women had weaker suicidal ideation, which is inconsistent with the results of a survey published in 2014 [8]. We also found that women made more suicide attempts than men. Future studies need to verify these findings and explore possible reasons by considering gender differences in the characteristics of college students. Furthermore, we found that men showed a full mediating effect of glorification/normalization, whereas women showed a partial mediating effect. The reasons for this remain unclear, and further investigating into gender differences in the glorification/normalization of suicide will help explain this finding.

This study has several limitations that future studies should pay more attention to. Firstly, the results of the mediation analyses were generated from a cross-sectional dataset. Using a longitudinal design is recommended in order to establish a causal relationship [74]. Future studies could use a multi-wave longitudinal study to establish a causal relationship. Secondly, the target population is college students, which may reduce the generalizability of the current findings to other populations who have characteristics quietly different from the age group of the current study (e.g., adolescents and the elderly). In a recent survey conducted in Shanghai, China, public stigma toward suicide was found to be greater in adolescent participants than in adults [43]. Therefore, it will be worthwhile to examine whether the findings of the current study can be generalized to this population. Thirdly, the impact of the COVID-19 pandemic remains unclear in relation to trends in the suicide of college students from which our participants were recruited. This pandemic has caused large-scale disruptions including depression, anxiety, and stress. Although recent studies have not observed an increase in suicide rates in the first months of the pandemic [75,76,77,78], it has been reported that disruptions caused by the pandemic, including depression and anxiety, are important factors associated with suicidal ideation and behavior in college students [72,73] and adults [79,80,81]. Future studies are needed to examine the long-term impact of the pandemic on suicide [75,76,77,82].

## 5. Conclusions

In this study, we examined the relationships between endorsement of suicide, stigma toward suicide, suicidal ideation, and suicide attempts among Chinese college students. Using mediation analysis, we found a partial mediating role of suicide ideation in the association between endorsement of suicide and suicide attempts and the association between stigma toward suicide and suicide attempts. Gender comparisons revealed a partial mediating effect in women and a full mediating effect in men for endorsement of suicide and revealed a significant suppression effect of suicidal ideation as a mediator between stigma toward suicide and suicide attempts in women. These results suggest that endorsement of suicide and stigma toward suicide are important factors that need to be seriously considered when examining the links between stereotypes toward suicide and the ideation-to-action framework of suicide. In the practice of suicide prevention, some strategies should be devised in order to reduce or eliminate the negative influences of endorsement of suicide and stigma toward suicide on suicidal ideation. Specifically, cultural and social factors associated with the endorsement of suicide should receive more attention in order to design preventive strategies. Future studies may consider exploring the generalizability of these findings to other populations, such as adolescents.

## Figures and Tables

**Table 1:** Participant distributions based on gender and education level.

Education Level	Men	Women
Year 1	5.51% (52)	11.02% (104)
Year 2	10.91% (103)	13.88% (131)
Year 3	16.53% (156)	18.64% (176)
Year 4	4.77% (45)	12.29% (116)
Graduate school	1.91% (18)	4.34% (41)
Total	39.62% (374)	60.38% (570)

Note. N = 944.

**Table 2:** Descriptive Statistics for the Scores on SOSS, SIDAS, and SBQ-R.

Variables	Mean (±SD)	Percentiles		
		25%	50%	75%
SOSS-Stig	2.59 (±0.93)	2.00	2.60	3.20
SOSS-I/D	3.84 (±0.87)	3.67	4.00	4.33
SOSS-G/N	2.64 (±0.98)	2.00	2.50	3.25
SIDAS	7.60 (±11.31)	0.00	0.00	12.00
SBQ-R	6.57 (±3.25)	4.00	6.00	9.00

Note. N = 944. SOSS-Stigma, stigma factor of the Stigma of Suicide Scale (SOSS). SOSS-I/D, isolation/depression factor of SOSS. SOSS-G/N, glorification/normalization factor of SOSS. SIDAS, the Suicidal Ideation Attribute Scale. SBQ-R, the Suicidal Behaviors Questionnaire-Revised.

**Table 3:** Intercorrelations of the Scores on SOSS, SIDAS, and SBQ-R.

Variables	1	2	3	4	5
1. SOSS-Stigma	—				
2. SOSS-I/D	0.34 *** [0.28, 0.39]	—			
3. SOSS-G/N	−0.13 *** [−0.21, −0.05]	−0.33 *** [−0.40, −0.26]	—		
4. SIDAS	0.22 *** [0.15, 0.29]	−0.06 [−0.12, 0.01]	0.38 *** [0.31, 0.44]	—	
5. SBQ-R	−0.09 ** [−0.16, −0.02]	0.12 *** [0.06, 0.18]	0.23 *** [0.17, 0.30]	0.49 *** [0.43, 0.54]	—

Note. N = 944. 95% confidence intervals based on 5000 times bootstrap were also provided. SOSS-Stigma, the stigma factor of the Stigma of Suicide Scale (SOSS). SOSS-I/D, the isolation/depression factor. SOSS-G/N, the glorification/normalization factor. SIDAS, the Suicidal Ideation Attribute Scale. SBQ-R, the Suicidal Behaviors Questionnaire-Revised. ** *p* < 0.01, *** *p* < 0.001 (two-tailed).

**Table 4:** Estimated mediating effect of suicidal ideation in the association between glorification/normalization of suicide and suicide attempts. Gender, age, and education level were added as covariates.

	Effect	Estimate	*p*	95% Confidence Interval
G/N	Direct effect	0.227	0.022	[−0.032, 0.423]
	Mediating effect	0.590	<0.001	[0.473, 0.705]
	Total effect	0.818	<0.001	[0.610, 1.025]
Stigma	Direct effect	−0.636	<0.001	[−0.827, −0.445]
	Mediating effect	0.376	<0.001	[0.225, 0.520]
	Total effect	−0.260	0.023	[−0.484, −0.037]

Note. Direct effect, glorification/normalization or stigma → suicide attempt. Mediating or indirect effect, glorification/normalization or stigma → suicidal ideation → suicide attempts. Total effect, the sum of the direct and mediating effects. Covariates: gender, age, education level.

**Table 5:** Means and Standard Deviations of each measure in men and women and the results of the *t*-tests for determining gender effects.

Measures	Women		Men		*t*	*p*	Cohen’s d
	Mean	SD	Mean	SD			
SOSS-G/N	2.59	0.91	2.71	1.08	1.73	0.084	0.120
SOSS-I/D	3.93	0.77	3.69	0.99	4.29	<0.001	0.271
SOSS-Stig	2.76	0.98	2.49	0.89	4.33	<0.001	0.288
SIDAS	6.58	10.35	9.16	12.49	3.46	<0.001	0.225
SBQ-R	6.88	3.32	6.10	3.07	3.61	<0.001	0.244

Note. N = 944, 570 women and 374 men. SOSS-G/N, glorification/normalization factor of SOSS. SIDAS, the Suicidal Ideation Attribute Scale. SBQ-R, the Suicidal Behaviors Questionnaire-Revised.

**Table 6:** Estimated mediating effect of suicidal ideation in the association between glorification/normalization and stigma toward suicide and suicide attempts in women and men. Age and education level were entered as covariates.

	Effect	Estimate	*p*	95% Confidence Interval
G/N Women	Direct effect	0.367	0.006	[0.107, 0.628]
	Mediating effect	0.634	<0.001	[0.462, 0.797]
	Total effect	1.002	<0.001	[0.708, 1.295]
G/N Men	Direct effect	0.116	0.433	[−0.174, 0.406]
	Mediating effect	0.497	<0.001	[0.342, 0.669]
	Total effect	0.612	<0.001	[0.321, 0.904]
Stigma Women	Direct effect	−0.799	<0.001	[−1.045, −0.552]
	Mediating effect	0.313	<0.001	[0.080, 0.526]
	Total effect	−0.485	0.002	[−0.792, −0.178]
Stigma Men	Direct effect	−0.352	0.020	[−0.648, −0.055]
	Mediating effect	0.372	<0.001	[0.193, 0.567]
	Total effect	0.021	0.901	[−0.303, 0.344]

Note. Direct effect, glorification/normalization or stigma → suicide attempt. Mediating or indirect effect, glorification/normalization or stigma → suicidal ideation → suicide attempts. Total effect, the sum of the direct and mediating effects.

## Data Availability

Data are available upon request.
